# Genetic Polymorphism of β-Casein Gene in Polish Red Cattle—Preliminary Study of A1 and A2 Frequency in Genetic Conservation Herd

**DOI:** 10.3390/ani9060377

**Published:** 2019-06-20

**Authors:** Anna Cieślińska, Ewa Fiedorowicz, Grzegorz Zwierzchowski, Natalia Kordulewska, Beata Jarmołowska, Elżbieta Kostyra

**Affiliations:** Department of Biochemistry, Faculty of Biology and Biotechnology, University of Warmia and Mazury in Olsztyn, Oczapowskiego 1A, 10-719 Olsztyn, Poland; ewa.kuzbida@uwm.edu.pl (E.F.); grzegorz.zwierzchowski@uwm.edu.pl (G.Z.); natalia.smulska@uwm.edu.pl (N.K.); bj58@wp.pl (B.J.); elzbieta.kostyra@uwm.edu.pl (E.K.)

**Keywords:** genetic variation, proteins, milk quality, milk casein, cows

## Abstract

**Simple Summary:**

Currently, milk and dairy products are considered nutritious foods because they contain bioactive constituents. However, in some cases, milk consumption might cause health-related issues, such as allergic reactions. Among all of the proteins present in milk, caseins deserve special attention. A genetic variant called β-casein is recognized as a potential cause of some health implications. However, although milk protein characteristics have been widely covered, a significant majority of published research focus on one cattle breed: Holstein There are numerous local breeds that have not been widely analyzed to date. One such breed is a dual-purpose breed called Polish Red.

**Abstract:**

Although there is growing interest in Red cow’s milk in Poland, to date there are few reports investigating the characteristics of milk components in the studied population. Particular emphasis on milk proteins is advised, since β-casein is a source of bioactive peptides named β-casomorphins. β-casomorphin 7, which originates mostly from β-casein variants A1, may be a significant risk factor in human ischemic heart disease, arteriosclerosis, type I diabetes, sudden infant death syndrome, and autism. The aim of the present study was to identify *CSN2* polymorphism gene in exon 7 using the genomic sequence from GenBank (M55158), g.8101C>A, (codon 67). Blood samples were collected from 201 Polish Red cattle (24 males and 177 females). The genotype of β-casein was determined using PCR-ACRS. The frequency of β-casein A2 in Polish Red population was 0.47. β-casein A2 frequency in Polish Red bulls and in cows was 0.58 and 0.37, respectively.

## 1. Introduction

The Polish Red (PR) breed population is increasing in numbers, although the most numerous dairy breed in Poland is Polish Holstein-Friesian (PHF). However, an increasing number of farmers are becoming more interested in the PR breed due to its high resistance and perfect adaptation to harsh environmental conditions. Moreover, PR is valued for its health, longevity, good fertility, and the high biological value of its milk. Due to the aforementioned benefits, PR was entered into the Genetic Resources Conservation Program, whose purpose is to preserve the genetic resources and diversity of native breeds and protect their gene pools [1]. Despite the fact that in Poland the PR is the breed mostly used as the meat type, the milk type head number is increasing in the overall PR population. Importantly, consumers are becoming more interested in PR milk due to its potential health benefits, which is encouraging a wider investigation of the characteristics of the milk produced from the studied population.

Proteins are one of the most important milk components, out of which caseins have received the greatest attention due to their recognized health-related properties. One of the major milk proteins is β-casein, and one of four casein milk proteins is particularly interesting. β-casein has 209 amino acid residues in its protein chain, and its gene (*CSN2*) belongs to the cluster of four casein genes located on chromosome 6 [2,3]. The most frequent genetic variants of *CSN2* are A1 and A2 [4,5]. The mutation that causes differences in the β-casein protein is a result of a single nucleotide polymorphism at codon 67 in exon 7 of the gene CCT (A2, proline) which results in CAT (A1, histidine) [2]. Bioactive peptide β-casomorphin 7 [5,6], which originates mostly from β-casein variants A1 [7,8,9], may be a significant risk factor in human ischemic heart disease, arteriosclerosis, type I diabetes, sudden infant death syndrome, and autism [5,10,11,12,13].

To our best knowledge, research has not yet been conducted on the frequency of bovine β-casein variants in PR. Therefore, we decided to analyze the frequency of the A1 and A2 allele of the β-casein gene in PR. In the presented work, our main aim was to identify *CSN2* polymorphism (GenBank M55158; g.8101C>A) gene within the exon 7 sequence. The experiment was conducted in a PR herd located in the north-east region of Poland.

## 2. Materials and Methods

### DNA Isolation and Genotyping

Blood samples were collected from 201 dual-purpose PR (24 bulls and 177 cows; born from 2001 to 2017) belonging to a herd located in the north-east region of Poland. All samples were collected by a veterinarian from the jugular vein. DNA was isolated from 200 µl of whole blood using GeneJET Whole Blood Genomic DNA Purification Mini Kit (Thermo Scientific, Waltham, MA, USA) according to the manufacturer’s instructions. The genotypes of β-casein locus (GenBank sequence acc. no. M55158)—A2 allele (CCT, GenBank: JX273429.1) and A1 allele (CAT, GenBank: JX273430.1) was determined using the PCR-ACRS (Amplification Created Restriction Site) described by Oleński et al. [14] with modifications. The contents in the 25-µL mixture consisted of DreamTaq Green Master Mix (Thermo Scientific), 150 ng of DNA, and molecularly pure water (Sigma–Aldrich, St. Louis, MO, USA), and starters with the following sequences:

CASB forward: 5’-GCAGAATTCTAGTCTATCCCTTCCCTGGACCCATGC-3’
CASB reverse: 5’-ACGGACTGAGGAGGAAACATGACAGTTGGAGGAAG-3’

PCR amplification was conducted in a thermal cycler according to the following program: initial denaturation: 94 °C for 3 min, proper denaturation: 94 °C for 30 s, attaching the starters at 62 °C for 30 s, synthesis: 72 °C for 30 s, final synthesis: 72 °C for 5 min, number of cycles: 40, cooling: 4 °C. The yield and specificity of the PCR products were evaluated after electrophoresis in 1.5% agarose gel (Promega, Madison, WI, USA) with GelGreen Nucleic Acid Gel Stain (Biotium, Fremont, CA, USA). To determine the genotype, restriction enzyme Mph1103I (NsiI, Thermo Scientific) was used and then electrophoresed in 2.5% agarose gel (Promega) with GelGreen Nucleic Acid Gel Stain (Biotium). Each A2A2 homozygote was confirmed a second time via a genotyping procedure. Genetic equilibrium of the examined population was estimated due to the Hardy–Weinberg principle and tested with the chi-square test (www.dr-petrek.eu/documents/HWE.xls). The study was conducted with compliance to local bioethics committee guidelines (18/2013).

## 3. Results and Discussion

The observed genotype frequencies of A1/A2 β-casein polymorphism conformed to the Hardy–Weinberg equilibrium. The frequency of β-casein A2 allele in the entire research Polish Red population was 0.47, which, according to a comparison of the frequencies of the *CSN2* allele in Red breeds in other European countries, was similar to the frequency observed in the Swedish PR population—0.51 [15]. The frequencies of the β-casein A1 and A2 alleles and genotypes in the research population are presented in Table 1. The obtained β-casein A2 frequency in research PR population was lower than the frequency observed in the Holstein-Friesian breed in Poland—0.6–0.65 [5,14,16], and in other European countries (Table 2). In PR cows, the A2-allele frequency was 0.37, and in bulls it was 0.58. The high frequency of the β-casein A2 allele in the tested bulls may be an effect of the small number of tested animals. However, it should be noted that bulls as donors of semen are the main source of the genetic pool.

Our results present the distribution of *CSN2* A2 and A1 alleles among PR cows and bulls in an analyzed herd from the north-east region of Poland. Because PR milk is characterized as having greater protein content, and consequently β- and κ-casein percentage [17], it might be considered as a relevant source of nutritional components for humans. On the contrary, the adverse effect of the β-casein A1 variant on human health has been widely discussed [18,19,20]. Moreover, New Zealand was the first country to have eliminated the A1 allele from its dairy cattle population, with no negative effect on milk yield and composition [5].

DNA-based genotyping is a fast and low-cost method. It allows for the β-casein A1 frequency to be monitored in dairy cattle in order to avoid the spread of an unfavorable allele in a population of cattle. Although a relatively high frequency of β-casein A1 allele was observed in PR cattle, it is important to note that a high turnout of the desired allele is observed in the bull population. Information on β-casein genotypes will allow for more conscious crossbreeding of animals and the elimination of an unfavorable allele from the population.

## 4. Conclusions

As the A2 beta-casein variant in milk is desirable in the population of cattle, the Polish Red breed, which is undergoing the reconstruction of the herd, provides a good potential for increasing this favorable allele through appropriate breeding of individuals. Moreover, the high turnout of the A2 beta-casein allele (0.58) among the red Polish bulls may increase its attendance among the entire population.

## Figures and Tables

**Table 1:** Genotype and allele frequency of β-casein in Polish Red cows and bulls.

Group	*N* (%) of β-Casein Genotypes			Frequency of Alleles		*p*-Value
	A1A1	A1A2	A2A2	A1	A2	
Cows	65 (36.7)	93 (52.5)	19 (10.7)	0.63	0.37	0.001
Bulls	3 (12.5)	14 (58.3)	7 (29.2)	0.42	0.58	0.001

**Table 2:** Occurrence of β-casein gene variants in Holstein-Friesian (HF) in Poland and Red in other countries (data sorted by increasing A1 allele frequency) (adapted from Kamiński et al., 2007 [5]).

Breed	Country	Allele Frequency of β-Casein		*N*	References
		A1	A2		
HF	Denmark	0.266	0.614	415	Gustavsson et al., 2014 [15]
	The Netherlands	0.28	0.50	1929	Visker et al., 2010 [21]
	The Netherlands	0.029	0.69	1629	Heck et al., 2009 [22]
	Poland	0.32	0.68	177	Cieślińska et al., 2012 [8]
	Poland	0.35	0.65	650	Oleński et al., 2012 [14]
	Thailand	0.363	0.602	231	Molee et al., 2011 [23]
	Italy	0.371	0.546	1226	Massella et al., 2017 [24]
	Italy	0.395	0.57	100	Chessa et al., 2013 [25]
	Poland	0.40	0.60	143	Kamiński et al., 2006 [16]
	China	0.432	0.459	133	Dai et al., 2016 [26]
	Turkey	0.485	0.456	49	Dinc et al., 2013 [27]
	Iran	0.50	0.50	119	Gholami et al., 2016 [28]
Red	Sweden	0.48	0.51	392	Gustavsson et al., 2014 [15]
	Denmark	0.71	0.23	169	Bech and Kristiansen, 1990 [29]
	Poland	0.53	0.47	201	Present data

Other variants of the β-casein gene are not included.
